# Atrial Dilated Cardiomyopathy: From Molecular Pathogenesis to Clinical Implications

**DOI:** 10.3390/jcm14248773

**Published:** 2025-12-11

**Authors:** Maria Cristina Carella, Marco Maria Dicorato, Vincenzo Ezio Santobuono, Ilaria Dentamaro, Paolo Basile, Stefania Piccolo, Antonio Labellarte, Michele Davide Latorre, Eduardo Urgesi, Gianluca Pontone, Nicoletta Resta, Eloisa Arbustini, Marco Matteo Ciccone, Andrea Igoren Guaricci, Cinzia Forleo

**Affiliations:** 1 Cardiovascular Disease Section, Interdisciplinary Department of Medicine (DIM), University of Bari “Aldo Moro”, University Hospital Consortium, Polyclinic of Bari, 70124 Bari, Italym.dicorato20@studenti.uniba.it (M.M.D.); vincenzoezio.santobuono@uniba.it (V.E.S.); ilaria.dentamaro.dott@gmail.com (I.D.); paolo.basile@uniba.it (P.B.); stefaniapiccolo18@gmail.com (S.P.); a.labellarte10@studenti.uniba.it (A.L.); micheledavide.latorre@policlinico.ba.it (M.D.L.); e.urgesi1@studenti.uniba.it (E.U.); marcomatteo.ciccone@uniba.it (M.M.C.); cinzia.forleo@uniba.it (C.F.); 2 Internal Medicine Section, Department of Precision and Regenerative Medicine and Ionian Area (DiMePRe-J), University of Bari “Aldo Moro”, University Hospital Consortium, Polyclinic of Bari, 70124 Bari, Italy; 3 Department of Perioperative Cardiology and Cardiovascular Imaging, IRCCS Centro Cardiologico Monzino, 20138 Milan, Italy; gianluca.pontone@cardiologicomonzino.it; 4 Department of Biomedical, Surgical and Dental Sciences, University of Milan, 20100 Milan, Italy; 5 Medical Genetics Unit, Department of Precision and Regenerative Medicine and Ionian Area (DiMePRe-J), University of Bari “Aldo Moro”, University Hospital Consortium, Polyclinic of Bari, 70124 Bari, Italy; nicoletta.resta@uniba.it; 6 Department of Research, Centre for Inherited Cardiovascular Diseases, IRCCS Foundation, University Hospital Policlinico San Matteo, 27100 Pavia, Italy; e.arbustini@smatteo.pv.it

**Keywords:** atrial dilated cardiomyopathy, natriuretic peptide A, atrial palsy, thromboembolism, atrial standstill

## Abstract

Atrial dilated cardiomyopathy with progression to atrial standstill is an ultrarare arrhythmogenic disorder characterized by complete loss of atrial electrical and mechanical activity. This condition, which may occur sporadically or in familial clusters, is associated with a markedly increased thromboembolic risk. The electrocardiographic hallmark is the absence of P waves combined with a bradycardic junctional escape rhythm. Biatrial enlargement gradually evolves into giant atria with preserved biventricular systolic function, while supraventricular arrhythmias and progressive atrial inexcitability dominate the clinical course. Valvular regurgitation frequently worsens in parallel with atrial remodelling, and patients often require permanent pacemaker implantation as well as lifelong anticoagulation. Among the few genetic determinants identified, the homozygous c.449G>A (p.Arg150Gln) mutation in the Natriuretic Peptide A gene represents one of the best characterized mechanisms. Disertori et al. first reported this pathogenic variant in 13 affected individuals from Italian families, establishing a recessive inheritance pattern. More recently, Silva et al. and Forleo et al. described additional cases, expanding the phenotypic spectrum of NPPA-related atrial cardiomyopathy. These findings confirm that homozygous carriers develop a severe atrial phenotype, whereas heterozygous relatives typically remain asymptomatic, underlining the importance of genetic testing in young patients with unexplained atrial fibrillation or standstill. Recognition of atrial cardiomyopathy as a distinct clinical entity is crucial, since early diagnosis may guide timely anticoagulation, arrhythmia management, and tailored follow-up. Broader adoption of genetic screening in patients with isolated atrial dysfunction could support precision medicine approaches, improve risk stratification, and ultimately prevent adverse outcomes in this ultrarare but highly morbid condition.

## 1. Introduction

Atrial standstill (AS), also referred to as “atrial silence” or “atrial paralysis,” represents one of the rarest forms of atrial cardiomyopathy [1]. It is defined by the complete absence of atrial electrical and mechanical activity, leading to loss of sinus rhythm and replacement by a slow junctional escape rhythm [1]. The atrial myocardium is both electrically silent and structurally unexcitable, with profound implications for conduction, hemodynamic, and thromboembolic risk.

Clinically, AS may occur sporadically or within families and can be idiopathic or secondary to conditions such as neuromuscular dystrophies, laminopathies, cardiac amyloidosis, or congenital anomalies like Ebstein’s malformation [1,2,3]. Its true incidence remains unknown, largely due to its rarity and the variable electrocardiographic presentations that may mimic more common atrial arrhythmias in early stages. Typical findings include the absence of P waves on surface electrocardiogram, low atrial voltage, and junctional escape rhythm, often leading to bradycardia and symptomatic conduction disturbances [1].

The idiopathic familial form of atrial dilated cardiomyopathy (ADCM) has attracted increasing attention in the last two decades. Different genetic backgrounds have been implicated: compound heterozygous mutations in *SCN5A* and *Connexin40*, recessive mutations in *SCN5A*, and—most notably—homozygous mutations in the *Natriuretic Peptide Precursor A* (*NPPA*) genes. Among these, the homozygous c.449G>A (p.Arg150Gln) variant in *NPPA* represents one of the best-characterized mechanisms [2]. Disertori et al. were the first to describe this mutation in a cohort of 13 patients, outlining a distinctive phenotype of progressive biatrial dilatation, supraventricular arrhythmias, atrial electrical silence, and preserved ventricular function [2].

More recent reports expanded the *NPPA*-related phenotypic spectrum. Silva et al. described a patient with fibrotic atrial cardiomyopathy and persistent atrial fibrillation (AF) carrying the same variant, while Forleo et al. reported the fifteenth documented case worldwide, reinforcing the concept of autosomal recessive inheritance and highlighting the clinical value of genetic testing in young individuals with unexplained atrial dysfunction [4,5].

Taken together, these findings establish NPPA-associated ADCM as a distinct and ultrarare genetic atrial cardiomyopathy. Its recognition is crucial, not only for accurate diagnosis and familial screening, but also for tailoring arrhythmia management and anticoagulation strategies to reduce the considerable risk of thromboembolic complications.

## 2. Methods

Given the exceptional rarity of atrial dilated cardiomyopathy associated with *NPPA* mutations, the available scientific evidence is limited to case reports, small family studies, and a few observational series. These sources, while valuable, are subject to important methodological constraints, including small sample sizes, potential ascertainment bias, and the absence of mechanistic or longitudinal data. As a result, many published observations cannot be generalized, and their conclusions must be interpreted with caution. Consequently, this review has been designed as a narrative synthesis rather than a systematic analysis.

A comprehensive literature search was conducted in PubMed/MEDLINE and Embase from 1946 to October 2025 using the terms “*atrial standstill*,” “*atrial dilated cardiomyopathy*,” “*NPPA*,” “*atrial natriuretic peptide*,” and “*atrial cardiomyopathy*”. Reference lists of all relevant articles were screened to identify additional contributions.

Approximately 120 records were initially retrieved and screened based on title and abstract. We included studies published in English that reported genetic, clinical, imaging, or pathophysiological aspects of primary atrial cardiomyopathy. Duplicates, conference abstracts without sufficient detail, and articles unrelated to atrial cardiomyopathy were excluded. Thus, rather than providing a chronological summary of previously published cases, we now present an integrated, thematic framework that synthesizes genetic, molecular, electrophysiological, and clinical evidence into a unified model of atrial-specific disease.

## 3. Discussion and State-of-the-Art

The first description of AS was reported by Chavez. From that initial observation until 1982, approximately 100 additional cases were described, and between 1982 and 2004, another 50 patients were reported [1,6].

In 1978, Woolliscroft and Tuna analyzed 55 patients with permanent atrial palsy, offering one of the earliest systematic classifications [3]. They divided patients into three clinical groups (Table 1).

1. Patients with long-standing heart disease and prior supraventricular arrhythmias. In this cohort, AS was considered the final stage of myocardial fibrosis. Patients often progressed from transient atrial paralysis, still responsive to atrial pacing, to irreversible standstill. Histological examination of right atrial (RA) biopsies revealed diffuse fibrosis, supporting the notion that atrial paralysis can represent a nonspecific outcome of advanced structural remodelling.
2. Patients with neuromuscular disorders. These included facioscapulohumeral muscular dystrophy [7,8,9], Charcot-Marie muscular dystrophy [10], an X-linked humeral-peroneal neuromuscular disease, Emery-Dreifuss muscular dystrophy (EDMD) [11], and limb-girdle muscular dystrophy [12,13]. Remarkably, Woolliscroft described the first case in which permanent AS preceded overt skeletal muscle involvement, highlighting the arrhythmia as an early manifestation [3]. Autopsy data in this subgroup confirmed diffuse atrial fibrosis and myocardial scarring.
3. Patients without prior cardiac or neuromuscular disease. In these cases, AS was discovered incidentally during evaluations for syncope, vertigo, or ischemic stroke, or at routine physical examination. Histological studies of RA and ventricular tissue revealed diffuse myocyte degeneration, interstitial expansion, and thickening of the RA endocardium [3].

**Figure 1:**
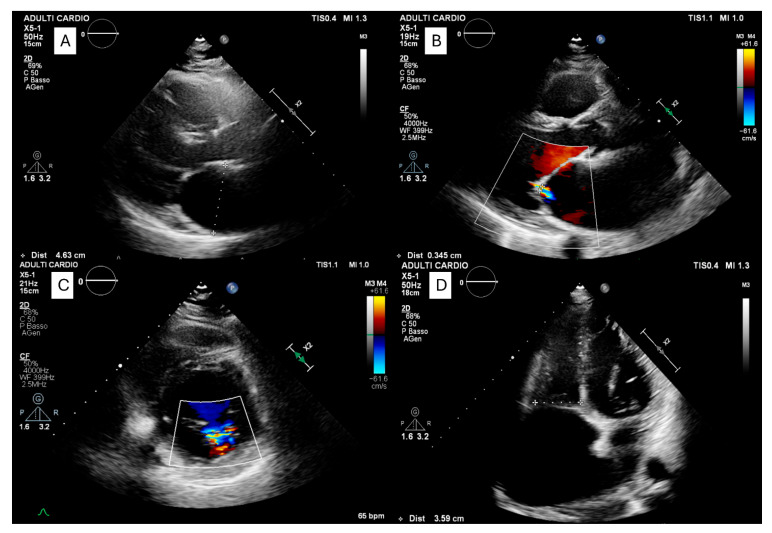
**Transthoracic echocardiographic findings in a patient carrying a homozygous variant in the *NPPA* gene.** Transthoracic echocardiography showing a parasternal long-axis view (**A**) with measurement of the left atrial anteroposterior diameter (46 mm). Image (**B**) demonstrates mild mitral regurgitation with a vena contracta of 3 mm. Panel (**C**) shows mitral regurgitation on the parasternal short-axis view. In the apical four-chamber view (**D**) focused on the right heart, severe right atrial enlargement is evident, with preserved right ventricular size.

In 1983, Disertori et al. described eight patients with persistent AS from three families in Trentino, Italy [14]. This was the first clear report of familial clustering. In the subsequent literature, only four additional groups described familial persistent AS, with no more than four affected members per family [15,16]. Building on these findings, Disertori and colleagues later identified a homozygous *NPPA* missense mutation (c.449G>A, p.Arg150Gln), defining a new entity termed ADCM [2,17]. The phenotype included massive biatrial dilatation, early-onset supraventricular arrhythmias, progressive atrial electrical silence, preserved left ventricular systolic function, and long-term functional stability (Figure 1).

Disease expression was shown to be age-dependent, and as atrial dilatation progressed, atrioventricular annuli enlarged, leading to worsening valvular regurgitation [2]. Thromboembolic complications were common, while advanced conduction disturbances often required pacemaker or implantable cardioverter-defibrillator therapy.

In 1991, Talwar et al. demonstrated that atrial paralysis may occasionally be confined to the RA, while the left atrium (LA) remains electrically active and stimulable [18]. In their series of four patients, endomyocardial biopsy of the right ventricle (when RA tissue was unavailable) revealed heterogeneous substrates including inflammatory myocarditis, amyloidosis, and hypertrophic fibrosis [18]. Despite pacemaker implantation, one patient with amyloidosis died suddenly, underlining the severity of this condition.

Further insights came from Nakazato et al., who studied 11 patients to clarify the clinical spectrum of AS [19]. Most had underlying structural heart disease, while only two were idiopathic. At admission, six patients lacked P waves, while others presented with atrial flutter, AF, or multifocal atrial tachycardia. Follow-up revealed progression toward ectopic atrial tachycardia or rhythms with progressively attenuated P waves until complete disappearance. Intracardiac mapping showed that atrial electrograms were first lost in the high and mid-lateral RA, spreading to the tricuspid annulus. Over 64 months, four patients died [19]. These findings suggested that atrial muscle injury follows a characteristic spatiotemporal progression and may involve both atrial myocardium and the atrioventricular conduction system, particularly in patients with myocarditis or dilated cardiomyopathy as underlying substrates.

Overall, AS evolved from a heterogeneous manifestation to a genetically and pathophysiologically defined entity, with NPPA-related ADCM representing a pivotal link between early clinical observations and modern genetic atrial cardiomyopathy concepts.

## 4. Classification and Etiology

Several attempts have been made to classify AS, often based on the extent of atrial electrical inactivity or the underlying etiologic substrate. The most widely accepted distinction relies on the degree and distribution of atrial electrical silence, as determined by intracardiac mapping.

- **Partial AS** refers to cases in which residual excitability persists in discrete regions of the RA [20,21]. Diagnosis requires multipoint atrial recordings, as standard surface electrocardiography may fail to detect localized activity. This form is often secondary to organic heart disease—including valvular lesions, ischemic cardiomyopathy, or infiltrative processes—and may remain clinically silent for years. In symptomatic patients with significant bradyarrhythmias or chronotropic incompetence, permanent pacing is generally indicated.
- **Total AS**, by contrast, is defined by the complete absence of excitable atrial tissue and failure to elicit any atrial response to pacing [20]. This form carries a more severe prognosis due to its association with extensive structural remodelling and higher thromboembolic risk.

From an etiological perspective, AS can be categorized as idiopathic or secondary. Idiopathic forms may occur sporadically or within families [22]. The familial idiopathic form is typically inherited in an autosomal dominant fashion with incomplete penetrance and has been associated with several gene variants affecting cardiac excitability and structural integrity [22].

Secondary forms may also appear sporadically or as part of genetically transmitted systemic or neuromuscular diseases. Reported aetiologies include Ebstein’s anomaly, chronic myocarditis, muscular dystrophies (especially EDMD, limb-girdle, and facioscapulohumeral variants), cardiac amyloidosis, ischemic heart disease, and diabetic cardiomyopathy [23,24,25,26]. Importantly, AS has also been described as an iatrogenic phenomenon, developing after catheter ablation for AF or extensive surgical atriotomies, likely due to iatrogenic atrial scarring and loss of excitability [27,28].

Based on the temporal evolution of the clinical phenotype, AS can be further classified into transient and persistent (or permanent) forms.

- The **transient form** is reversible and typically associated with acute conditions such as myocardial ischemia or infarction, myocarditis, electrolyte disturbances (particularly hyperkalaemia), hypoxia, digitalis or quinidine toxicity, electrical cardioversion, or recent cardiac surgery [29]. In these settings, atrial paralysis is a functional consequence of transient metabolic or inflammatory insults to the atrial myocardium rather than irreversible structural damage.
- The **persistent or permanent form** reflects chronic and irreversible loss of atrial excitability. It may arise idiopathically or in association with rheumatic heart disease, infiltrative cardiomyopathies, amyloidosis, diabetes mellitus, Ebstein’s anomaly, and hereditary neuromuscular disorders [1]. The electrocardiographic recognition of atrial paralysis now mandates the exclusion of EDMD, in which atrial arrhythmogenic pathology often precedes the clinical onset of skeletal muscle involvement.

This multidimensional classification, encompassing anatomical, etiologic, and temporal criteria, highlights the heterogeneity of AS and its overlap with broader categories of genetic and acquired atrial cardiomyopathies. Integrating genetic testing, multimodality imaging, and electrophysiological evaluation is therefore essential to refine diagnosis, guide management, and identify familial forms with prognostic implications [30,31].

## 5. Pathophysiology and Genetic Background

The pathophysiology of ADCM reflects the convergence of genetic, molecular, and hemodynamic factors, which collectively disrupt atrial homeostasis.

At the cellular level, the atrial myocardium exhibits unique electrophysiological and metabolic features that render it particularly vulnerable to injury. Chronic hemodynamic overload, oxidative stress, inflammation, and neurohormonal activation induce myocyte apoptosis, interstitial fibrosis, and gap junction remodelling, leading to conduction heterogeneity and atrial mechanical failure [32,33,34]. These processes are amplified in the presence of pathogenic variants affecting structural or ion channel proteins, where the genetic substrate acts as a primary driver rather than a secondary modifier of disease. From a genetic standpoint, several monogenic forms of atrial cardiomyopathy have been described, spanning a wide array of genes involved in electrical conduction, cytoskeletal integrity, and intercellular coupling (Table 2).

### 5.1. LMNA, DES, and EMD Gene Variants

AS can also occur as part of the arrhythmic spectrum of familial cardiomyopathies, particularly those characterized by early conduction system disease and progressive atrial electrical failure. Several disease-causing genes have been identified in these contexts, including *LMNA* (*lamin A*/*C*)—typically associated with dilated cardiomyopathy (DCM)—and *DES* (*desmin*), more commonly linked to restrictive, arrhythmogenic or mixed phenotypes. Additional genetic loci on chromosomes 2, 3, and 6 have also been implicated in familial forms of AS and atrial conduction disease [35,36].

Mutations in nuclear envelope genes—particularly *LMNA* and *EMD* (encoding *emerin*)—are the molecular hallmark of EDMD [35]. Emerin is a nuclear membrane protein belonging to the lamin-associated protein family and plays a crucial role in maintaining nuclear architecture and mechanotransduction [37]. While classical EDMD presents with early joint contractures and progressive skeletal muscle weakness, cardiac manifestations—especially atrial arrhythmias and conduction disease—often precede neuromuscular symptoms. Importantly, in 2020, Ishikawa et al. identified three X-linked recessive *EMD* mutations (start-loss, splicing, and missense) in families exhibiting a distinct nonsyndromic cardiac phenotype characterized by progressive atrial arrhythmias culminating in AS and left ventricular noncompaction (LVNC), but without the skeletal muscle features typical of EDMD [35]. All probands developed biatrial electrical standstill and progressive LVNC, and several family members suffered recurrent ischemic strokes. The strong familial clustering of thromboembolic events was attributed to the combination of atrial paralysis and reduced ventricular contractility. These findings expanded the clinical spectrum of *EMD*-related disease, demonstrating that emerin deficiency may cause not only classical EDMD with secondary AS but also isolated cardiac emerinopathy—a nonsyndromic, X-linked, progressive form of atrial standstill associated with LVNC and high thromboembolic risk.

### 5.2. SCN5A and RYR2 Gene Variants

Cases of familial idiopathic atrial palsy are extremely rare. Genetic variant in the *SCN5A* gene, which encodes the alpha subunit of the cardiac sodium channel protein Nav1.5, is well known as a cause of various inherited arrhythmic syndromes, including long QT syndrome type 3, Brugada syndrome (BrS), paroxysmal ventricular fibrillation, progressive familial heart block, AF, and sick sinus syndrome [38,39,40]. Loss-of-function or gain-of-function changes in cardiac sodium channel gating (such as Nav1.5) could also cause AS [41,42,43]. A cardiac sodium channel mutation *SCN5A* (*p.D1275N*) that co-segregated with a rare connexin 40 genotypes [at nucleotides −44 (G → A) and +71 (A → G)] has been documented in a family with AS [22]. A defect in the cardiac sodium channel gene *SCN5A* and two polymorphisms in the gene encoding a specific atrial gap junction protein, *connexin 40* (*Cx40)*, were identified in 3 living patients [22]. The authors suggest that the isolated functional effect of each individual mutation would be “phenotypically” benign, whereas the combined effect (*SCN5A* + *Cx40*) could play an important role in the genesis and progression of the disease [22]. In 2005, Makita et al. found a novel *SCN5A* mutation (*L212P*) in a proband with progressive atrial dysfunction leading to atrial arrest [44]. Unlike the relatively benign *D1275N* abnormalities, *L212P* showed severe dysfunction when expressed mutated [44]. In addition to *L212P*, the proband carried the maternal allele *Cx40* SNPs in regulatory regions. In contrast, the proband’s father, who carried the *L212P* allele but not the *Cx40* SNPs, was phenotypically normal [44].

Recently, Kato et al. found another missense mutation *S910L* (*c.2729c*>*t*), located in exon 16 of *SCN5A*, in a 4-year-old girl [45]. This was the first described case of AS developed in a child with histologically proven atrial fibrosis in association with an *SCN5A* mutation [45].

Based on this finding, Howard et al. published a retrospective multicenter study of pediatric patients at the time of diagnosis of AS (defined as absence of atrial activity documented during an electrophysiologic study, placement of a device, or noninvasive rhythm tracings and confirmed by an echocardiogram) [46]. Genetic testing identified *SCN5A* variants in 13 patients (65%) [46]. Ventricular arrhythmias and cardiac arrest were more commonly observed in patients with biallelic *SCN5A* variants [46]. The study authors concluded that AS may be associated with *SCN5A* variants with loss of function and that patients may experience atrial and ventricular arrhythmias and may present with problems during device placement; in addition, patients without atrial pacing capability are at risk for thromboembolic events and deserve anticoagulation [46].

Finally, a homozygous *V1340L* mutation in *SCN5A* was identified in a family with atrial stunting and sudden death [40]. Heterozygous mutations were identified in the proband’s mother, father, and brother. All three had normal sinus rhythm and were asymptomatic, demonstrating that only the homozygous mutation causes the phenotype [40].

A particularly intriguing association has emerged with *RYR2* mutations, which are classically related to catecholaminergic polymorphic ventricular tachycardia (CPVT) and arrhythmogenic right ventricular cardiomyopathy (ARVC). Deletions or loss-of-function variants in *RYR2*, which encodes the cardiac ryanodine receptor responsible for sarcoplasmic reticulum calcium release, can give rise to broader phenotypic spectra encompassing sinoatrial and atrioventricular node dysfunction, atrial fibrillation, AS, and even dilated cardiomyopathy [47]. This expanded phenotype underscores the central role of calcium-handling abnormalities in the pathogenesis of atrial electrical failure.

### 5.3. NPPA Gene Variant

Another known mutation that causes AS is that of the *NPPA* gene. It was identified in 2013 by Disertori et al. and has been confirmed in only 15 patients worldwide [2,4,5,17]!

In 1983 they had already described an 8-year follow-up of atrial dilatation with evolution to standstill in 8 patients [14]. They later identified 5 more patients with identical phenotypes: all families were from the same geographic area in northeastern Italy [14]. They monitored the evolution of the phenotypes, traced the natural history of idiopathic atrial dilatation with progression to AS, and identified a genetic association of the disease with a homozygous mutation in the natriuretic peptide A (*NPPA*) precursor gene [14]. They followed the 13 patients for 37 years. The disease was characterized by (1) clinical onset in adulthood; (2) biatrial dilatation to giant size; (3) early supraventricular arrhythmias with progressive loss of atrial electrical activity to atrial arrest; (4) thromboembolic complications; (5) stable and normal left ventricular function [14]. By sequencing the natriuretic peptide precursor A, they identified a homozygous missense mutation (*p.Arg150Gln*) in all living affected individuals in the 6 families [2,17]. Proatrial natriuretic peptide (MR-proANP) levels were markedly reduced in individuals homozygous for the mutant alleles. Heterozygous carriers of the mutation were healthy and showed normal levels of atrial natriuretic peptide [2,17]. The researchers concluded that autosomal recessive ADCM is a rare disease associated with a homozygous mutation in the *NPPA* precursor gene and characterized by extreme atrial dilatation with stationary evolution, thromboembolic risk, preserved left ventricular function, and severely reduced levels of atrial natriuretic peptide [2,17].

More recently, additional reports have expanded the clinical spectrum associated with the NPPA *p.Arg150Gln* mutation. In 2023, Silva Cunha et al. described a young man with early-onset AF, extensive biatrial fibrosis demonstrated by cardiac magnetic resonance and invasive electroanatomical mapping, and no structural ventricular disease [5]. Genetic testing confirmed a homozygous *NPPA p.Arg150Gln* variant, while both parents were heterozygous and asymptomatic, further supporting the autosomal recessive inheritance of this atrial-specific phenotype. In 2025, Forleo et al. reported the fifteenth known case worldwide, involving a woman with progressive atrial dilatation, atrial standstill, and preserved ventricular function. The case reinforced the concept of NPPA-related atrial dilated cardiomyopathy as a distinct fibrotic atrial disorder with high thromboembolic risk and normal ventricular mechanics [4].

Together, these recent reports consolidate the link between *NPPA* homozygous mutations and isolated atrial cardiomyopathy characterized by progressive fibrosis, atrial electrical standstill, and preserved ventricular function, expanding the landscape of this ultrarare genetic disease.

### 5.4. MYL4 Gene Variant

Recently, other mutations have been described, in the genes coding for Myosin Light Chain 4 (*MYL4*), which encodes the atrial-specific myosin essential light chain [35,48,49]; these mutations could be related to AS, but also to other familial diseases (such as AF, LVNC, and other neuromuscular disorders), as described by Gollob [48]. Peng et al. [50] described a large pedigree in which the pattern of inheritance was most likely autosomal dominant. They concluded that *MYL4* gene variants with loss of function cause progressive atrial cardiomyopathy in humans and rats, recognizing *MYL4* as a key gene required for contractile, electrical, and structural integrity of the atrium [50].

Common to all genetic forms of AS (and particularly in the forms caused by *SCN5A*, *NPPA*, *EMD*, and *MYL4*) is the inability to measure electrical signals from atrial tissue and the inability to electrically capture and contract atrial myocardium during electrophysiological study in advanced stages of the disease. Loss-of-function mutations in *EMD* and *MYL4* that affect their actin-binding interaction and impair Z-disc formation and T-tubule structure may explain the profound loss of excitation-contraction coupling observed in some patients carrying mutations in these genes [48]. Sodium channel mutations that have a unique depolarizing shift effect in channel activation kinetics, essentially creating a higher threshold for channel opening, may explain the presence of electrical stasis related to specific *SCN5A* variants [48]. When comparing the available genetic forms of ADCM, it becomes apparent that each gene can be associated with distinctive clinical trajectories, yet no genotype can be considered inherently less severe, as life-threatening complications—including sudden cardiac death—may occur even in the absence of overt ventricular dysfunction. *LMNA* variants tend to display the broadest systemic impact, with early conduction disease and a high risk of ventricular arrhythmias and sudden death. *NPPA* variants, by contrast, show a remarkably homogeneous and atrial-restricted profile characterized by biatrial dilation, early atrial standstill, and preserved ventricular function, supporting the notion that NPPA-related ADCM may represent a partially distinct clinical entity; however, these patients remain at substantial thromboembolic risk. Overall, genotype–phenotype correlations may help refine surveillance and management strategies.

## 6. Role of Atrial Natriuretic Peptide (ANP) in Atrial Dilated Cardiomyopathy

Disertori et al. first provided evidence of a genetic atrial cardiomyopathy characterized by a progressive fibrotic atrial substrate leading to a spectrum of supraventricular arrhythmias and culminating in AS [2]. This entity represents a paradigm of fibrotic atrial cardiomyopathy caused by molecular alterations in the Atrial Natriuretic Peptide (ANP) pathway, linking atrial mechanical dysfunction to electrical instability and structural remodelling.

ANP, a 28–amino acid peptide hormone, is synthesized and secreted predominantly by atrial cardiomyocytes, where it is stored as pro-ANP within dense granules. Under physiological conditions, its release is triggered by atrial wall stretch, whereas under pathological conditions, such as pressure or volume overload, heart failure, or ventricular hypertrophy, enhanced secretion of ANP serves as a compensatory mechanism to counteract increased atrial stress [51,52,53]. Once released into the circulation, ANP binds to its receptor NPRA (NPR1), activating a cyclic GMP (cGMP)–dependent signalling cascade that exerts natriuretic, diuretic, and vasodilatory effects [51,53,54]. Through these pathways, ANP regulates intravascular volume, arterial pressure, and vascular tone, and influences electrophysiological and structural properties of the atria.

Experimental studies have demonstrated that ANP modulates atrial conduction and refractoriness by enhancing intra-atrial conduction velocity and shortening the effective refractory period of the RA [2,51]. Conversely, ANP-deficient mice develop systemic hypertension, left ventricular hypertrophy, and an exaggerated hypertrophic response to pressure overload, accompanied by increased expression of pro-inflammatory cytokines and profibrotic mediators [55,56]. These findings indicate that, beyond its well-established antihypertensive actions, ANP also exerts direct cardioprotective effects on both atrial and ventricular myocardium, modulating hypertrophic signalling, fibrosis, and electrophysiological remodelling.

However, the specific role of ANP in atrial homeostasis and the molecular basis of ANP-related atrial disease remain incompletely understood. Both excess and deficiency of ANP may disturb myocardial signalling and promote atrial remodelling, providing mechanistic explanations for the variable phenotypes associated with *NPPA* mutations.

## 7. Diagnosis, Management and Prognosis

**Figure 2:**
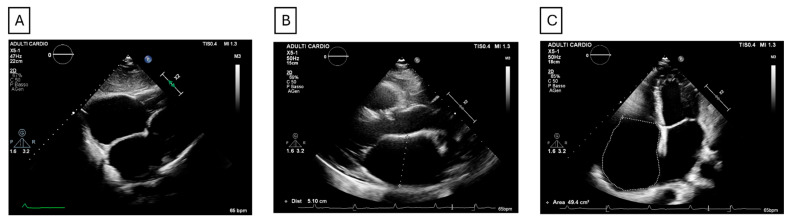
**Echocardiographic images representative of atrial cardiomyopathy.** (**A**) Subcostal view showing massive atrial enlargement and absence of pericardial effusion. (**B**) Parasternal long-axis view demonstrating left atrial dilation (antero-posterior diameter 51 mm). (**C**) Apical four-chamber view showing a right atrial area of approximately 50 cm^2^.

The diagnosis of permanent AS is important because of its prognostic implications. The absence of P waves on surface ECG and the presence of a junctional rhythm raises strong suspicion of atrial palsy [1,2]. Echocardiography provides a non-invasive means of demonstrating atrial stasis, documenting bilateral atriomegaly, absence of A wave on transmitral and transtricuspid Doppler flow, and mitral and tricuspid regurgitation [1,2] (Figure 2).

ANP assay may assist diagnosis. These markers, although informative, are not diagnostic determinants [1,2]. If noninvasive studies suggest permanent atrial stasis, invasive confirmation is required. The gold standard is the intracavitary electrophysiological study [1,14]. Several criteria have been proposed for diagnosis, but only mapping and pacing allow for distinction between partial and total forms [1,14]. The hallmark of total AS is absence of atrial electrical activation at all recording sites; in partial forms, this occurs only in certain areas [1,14]. Similarly, there is no electrical response to high-voltage stimulation at the same sites; electrophysiological study is conducted according to the classical technique (recording and pacing in the RA and coronary sinus for the LA) verifying the absence of biatrial electrical activity [1,14]. Usually, the dominant rhythm is a bradycardic junctional substitution rhythm.

In terms of clinical aspects, AS is characterized by an extremely heterogeneous symptom picture; most patients are asymptomatic and often come to the cardiologist’s observation occasionally as part of diagnostic procedures for other reasons. Palpitations, fainting, and Adams-Stokes episodes are the most common and nonspecific symptoms, while dyspnea, exercise intolerance, and progressive worsening of functional class are variously represented and depend on the type and degree of underlying heart disease [1]. In atrial palsy associated with muscle and neuromuscular disorders, the cardiac disorder may precede the musculoskeletal one [1]. Thromboembolic phenomena are a serious and very frequent complication [1]. These complications may be the first clinical manifestations that bring the patient to observation and may also be the cause of sudden cardiac death. Recent population-based analyses have confirmed that atrial cardiomyopathy, defined by structural and electrical atrial remodelling, represents a shared substrate for AF, heart failure and stroke, even in the absence of overt arrhythmia [57].

Management of ADCM and AS requires a tailored approach integrating genetic background, arrhythmic burden, thromboembolic risk, and the degree of atrial structural remodelling. Although no disease-specific guidelines exist, several principles can support clinical decision-making.

**Follow-up and Imaging.** In clinically stable patients, transthoracic echocardiography every 12 months seems to be appropriate to monitor atrial size, valve regurgitation, and ventricular function. Patients with progressive atrial dilation, new-onset arrhythmias, or high-risk genotypes (e.g., *LMNA*, *EMD*, *SCN5A*) may benefit from imaging every 6 months. Cardiac MRI can be repeated every 2–3 years to assess atrial fibrosis, identify early ventricular involvement, and guide long-term risk stratification.

**Rhythm and Conduction Monitoring.** Annual 24–48 h Holter monitoring is reasonable for patients without symptoms or conduction abnormalities, whereas individuals with syncope, palpitations, neuromuscular involvement, or high-risk genetic variants should undergo more frequent or prolonged ECG monitoring, including event recorders or implantable loop recorders when clinically indicated. Loss of atrial capture or progressive sinus node disease requires timely evaluation for permanent pacing.

**Anticoagulation Strategy.** Given the markedly elevated thromboembolic risk—even in the absence of atrial fibrillation—lifelong oral anticoagulation is mandatory [1]. Although comparative trials are lacking, NOACs are generally preferred over warfarin because of their more favorable safety profile, predictable pharmacokinetics, and lower risk of intracranial bleeding. Warfarin may be considered in patients with mechanical valves, significant chronic kidney disease, or specific contraindications to NOACs.

**Pacing Therapy.** Most patients eventually require implantation of a permanent pacemaker due to junctional bradycardia or advanced conduction disease. Ventricular lead placement may be challenging because of annular dilation and tricuspid regurgitation [58,59]. While traditional right ventricular apical pacing has been used, newer physiological strategies such as left bundle branch area pacing may improve long-term synchronization and hemodynamics [60].

**Heart Failure and Valvular Management.** Diuretics and vasodilators may be used according to symptoms and the degree of functional mitral or tricuspid regurgitation [1].

The prognosis of persistent atrial palsy varies depending on the clinical scenario in which it fits. In forms associated with cardiomyopathy or neuromuscular disorder, the prognosis is largely determined by the underlying disease, whereas in idiopathic forms they show a very slow prognosis [1].

## 8. Conclusions

AS encompasses a heterogeneous group of conditions resulting from diverse genetic and acquired causes. Among these, homozygous *NPPA* mutations define an ultrarare form of ADCM, providing crucial insight into the molecular basis of atrial electrical and structural failure. Recognition of NPPA-related disease, although exceptional, underscores the importance of genetic testing and tailored management in patients with unexplained atrial dysfunction. Future research should further elucidate ANP-related pathways and identify novel therapeutic targets for this and other forms of atrial cardiomyopathy.

## Figures and Tables

**Table 1:** Classification and causes of Atrial Standstill.

Type	Subtype	Etiology	Main Features
Primary (Idiopathic)	Familial/Sporadic	SCN5A, NPPA, EMD, LMNA, MYL4, RYR2	Biatrial, progressive, may be isolated
Secondary (Acquired)	Myocarditic, Infiltrative, Postsurgical	Amyloidosis, Ebstein’s anomaly, post-ablation, postoperative scarring	Partial or total, reversible or permanent
Neuromuscular	EDMD, muscular dystrophies	*LMNA*, *EMD*	Cardiac involvement may precede skeletal muscle manifestations

**Table 2:** **Genes associated with genetic forms of Atrial Standstill**.

Gene	Protein	Pathogenic Mechanism	Inheritance Pattern	Phenotypic Features	Genotype–Phenotype Correlations Relevant to Management
*NPPA*	Atrial natriuretic peptide (ANP)	Reduced secretion and atrial fibrosis	Autosomal recessive	Biatrial, progressive, preserved ventricular function	Very high thromboembolic risk even without AF → early anticoagulation; regular imaging to monitor fibrotic progression
*SCN5A*	Sodium Voltage-Gated Channel Alpha Subunit 5	Loss-of-function variants	Autosomal dominant or recessive	Atrial/ventricular arrhythmias, early-onset AS	Monitor for ventricular arrhythmias; anticipate device-implant challenges; consider anticoagulation when atrial capture is absent
*EMD*	Emerin	Nuclear envelope defect	X-linked recessive	AS with LVNC, thromboembolic strokes	Family screening; early anticoagulation; surveillance for LVNC and pacing issues
*LMNA*	Lamin A/C	Nuclear envelope defect	Autosomal dominant	AS in the context of laminopathy or DCM: high risk of ventricular arrhythmias, atrioventricular block, and sudden cardiac death	Close rhythm monitoring, frequent ECG Holters; low threshold for ICD due to malignant arrhythmic risk
*MYL4*	Myosin light chain 4	Impaired sarcomeric integrity	Autosomal dominant	Familial AF, progressive AS	Follow disease progression; evaluate for AF and loss of atrial mechanical function
*RYR2*	Ryanodine receptor 2	Abnormal calcium handling	Autosomal dominant	Catecholaminergic arrhythmias, sinoatrial and atrioventricular node dysfunction, atrial fibrillation, rare AS cases	Avoid adrenergic triggers; rhythm control; monitor for ventricular arrhythmias

## Data Availability

Data sharing not applicable.

## Abbreviations

The following abbreviations are used in this manuscript:

ADCM	Atrial dilated cardiomyopathy
AF	Atrial fibrillation
ARVC	Arrhythmogenic right ventricular cardiomyopathy
ANP	Atrial natriuretic peptide
AS	Atrial standstill
BrS	Brugada syndrome
Cx40	*Connexin40*
CPVT	Catecholaminergic polymorphic ventricular tachycardia
EDMD	Emery-Dreifuss muscular dystrophy
EMD	*Emerin*
DCM	Dilated Cardiomyopathy
DES	*Desmin*
LA	Left atrium
LMNA	*Lamin A*/*C*
LVNC	Left ventricular noncompaction
MYL4	*Myosin Light Chain 4*
NPPA	Natriuretic Peptide Precursor A
RA	Right atrium
RYR2	*Ryanodine receptor 2*
SCN5A	Sodium voltage-gated channel alpha subunit 5
SNPs	Single nucleotide polymorphisms
